# Whole transcriptomic analysis of mesenchymal stem cells cultured in Nichoid micro-scaffolds

**DOI:** 10.3389/fbioe.2022.945474

**Published:** 2023-01-06

**Authors:** Carolina Testa, Stefania Oliveto, Emanuela Jacchetti, Francesca Donnaloja, Chiara Martinelli, Pietro Pinoli, Roberto Osellame, Giulio Cerullo, Stefano Ceri, Stefano Biffo, Manuela T. Raimondi

**Affiliations:** ^1^ Department of Electronics, Information and Bioengineering, Politecnico di Milano, Milano, Italy; ^2^ Department of Chemistry, Materials and Chemical Engineering “Giulio Natta”, Politecnico di Milano, Milano, Italy; ^3^ Department of Bioscience (DBS), University of Milan, Milano, Italy; ^4^ Institute of Photonics and Nanotechnology (IFN)-CNR and Department of Physics, Politecnico di Milano, Milano, Italy

**Keywords:** mechanobiology, mesenchymal stem cells, synthetic, niche, 3D cell culture, transcriptomic analysis, cell adhesion molecules, bioengineering, immunemodulation

## Abstract

Mesenchymal stem cells (MSCs) are known to be ideal candidates for clinical applications where not only regenerative potential but also immunomodulation ability is fundamental. Over the last years, increasing efforts have been put into the design and fabrication of 3D synthetic niches, conceived to emulate the native tissue microenvironment and aiming at efficiently controlling the MSC phenotype *in vitro*. In this panorama, our group patented an engineered microstructured scaffold, called Nichoid. It is fabricated through two-photon polymerization, a technique enabling the creation of 3D structures with control of scaffold geometry at the cell level and spatial resolution beyond the diffraction limit, down to 100 nm. The Nichoid’s capacity to maintain higher levels of stemness as compared to 2D substrates, with no need for adding exogenous soluble factors, has already been demonstrated in MSCs, neural precursors, and murine embryonic stem cells. In this work, we evaluated how three-dimensionality can influence the whole gene expression profile in rat MSCs. Our results show that at only 4 days from cell seeding, gene activation is affected in a significant way, since 654 genes appear to be differentially expressed (392 upregulated and 262 downregulated) between cells cultured in 3D Nichoids and in 2D controls. The functional enrichment analysis shows that differentially expressed genes are mainly enriched in pathways related to the actin cytoskeleton, extracellular matrix (ECM), and, in particular, cell adhesion molecules (CAMs), thus confirming the important role of cell morphology and adhesions in determining the MSC phenotype. In conclusion, our results suggest that the Nichoid, thanks to its exclusive architecture and 3D cell adhesion properties, is not only a useful tool for governing cell stemness but could also be a means for controlling immune-related MSC features specifically involved in cell migration.

## 1 Introduction

Traditionally, the most utilized substrates for cell culture have been treated, polystyrene or glass surfaces (Kapałczyńska et al., 2016), but since the environment provided by these systems is generally flat, cells grow forming monolayers, consequently impairing cell–cell and cell–ECM interactions and also modifying their morphology with respect to their native configuration. In this way, cells shape their cytoskeleton, sending different messages to the nucleus compared to the physiological state (Von der Mark et al., 1977; Petersen et al., 1992; Debnath and Brugge, 2005; Nelson and Bissell, 2006; Mahmud et al., 2009; Kilian et al., 2010), thus altering gene expression, protein synthesis, and other cell functions (Greiner et al., 2012; Santos et al., 2012). For these reasons, over the last few decades, three-dimensional (3D) systems for *in vitro* cell culture have gained increasing interest in several fields of biological research. It has been largely demonstrated that 3D culture conditions constitute a more realistic model as compared to bidimensional (2D) systems, since they create a more physiological microenvironment and, thus, promote cell responses that are more similar to the *in vivo* ones (Pampaloni et al., 2007; Edmondson et al., 2014; Shamir and Ewald, 2014).

One of the areas of greatest interest for the employment of 3D culture systems is stem cell research for tissue engineering and regenerative medicine, therapeutical approaches based on the use of stem cells to repair and regenerate damaged organs and tissues in place of resorting to allogenic transplantation (Heidary Rouchi and Mahdavi-Mazdeh, 2015). This is possible thanks to the self-renewal capacity of stem cells, the ability to maintain their stemness while dividing, and their ability to differentiate toward specific lineages under precise conditions (Kolios and Moodley, 2012; Dulak et al., 2015). The therapeutic potential of different stem cell types has been investigated, and adult stem cells and mesenchymal stem cells, in particular, have displayed the highest potential (Dulak et al., 2015; Liu, 2017). Mesenchymal stem cells (MSCs) are multipotent adult stem cells that can be easily isolated from the bone marrow (BM), adipose tissue, or umbilical cord and are able to differentiate into adipocytes, chondrocytes, osteoblasts, myocytes, smooth muscle cells, and neuron-like cells (Almalki and Agrawal, 2016; Liu, 2017; McKee and Chaudhry, 2017; Saidova and Vorobjev, 2020). Physiologically, MSCs and adult stem cells, in general, reside in specific “niches” which not only provide an anatomical location but also support self-renewal and stemness maintenance through biochemical and biophysical cues (Morrison and Spradling, 2008; Walker et al., 2008). In the last decade, increasing efforts have been made in designing and fabricating 3D synthetic niches, aiming to efficiently control MSCs’ fate *in vitro* and produce therapeutic cells on a large scale (Joddar and Ito, 2013). These scaffolds are conceived to emulate the native microenvironment by finely tuning the substrate physical properties, such as nanotopography (Dalby et al., 2007; Engel et al., 2009; McMurray et al., 2011), material stiffness (Engler et al., 2006; Khatiwala et al., 2009; Winer et al., 2009), and microgeometry (X. Li et al., 2012; Naito et al., 2011; Nerurkar et al., 2011). In the panorama of 3D cell culture systems for stem cell expansion, an innovative engineered substrate mimicking the native stem cell niche, called Nichoid, has recently been developed. It is based on an elementary and easily reproducible microarchitecture. Indeed, it is composed of a 3D interconnection of grids and columns able to create perfectly defined pores at the micrometric scale (Ricci et al., 2017). The main peculiarity of this scaffold is the use of two-photon polymerization (2PP) as a fabrication method, a technique enabling the creation of 3D structures based on a computer-generated model, with control of scaffold geometry at the cellular level (10 μm) and spatial resolution beyond the diffraction limit, up to 100 nm (Wu et al., 2006; Malinauskas et al., 2010; Nguyen and Narayan, 2017). It is known from the literature that isotropic cytoskeletal forces promote self-renewal and pluripotency, together with low extracellular loads and low oxygen concentration (McBeath et al., 2004; Guilak et al., 2009; Gilbert et al., 2010; Wan et al., 2010; Nava et al., 2012). The Nichoid substrate was conceived with a 3D microtopology capable of exerting an isotropic system of adhesion forces on cells while reducing cytoskeletal tension. The pore microgeometry is proven to promote stem cell homing inside the structure (Raimondi et al., 2013). Therefore, the fundamental property reproduced on cells by the Nichoid scaffold is the capability to interact mechanically with cells at the single-cell scale, thanks to the very high spatial resolution of the technique used for its microfabrication. The “niche effect” has been investigated by our group on murine embryonic stem cells (mESCs), human and rat bone marrow MSCs, and murine neural precursor cells (NPCs); all these studies have highlighted the Nichoid’s ability to maintain stemness and pluripotency genes switched on at higher levels compared to traditional flat substrates, with no need for adding exogenous soluble factors or feeder layers (Nava et al., 2016a; Nava et al., 2016b; Bonandrini et al., 2018; Carelli et al., 2020; Remuzzi et al., 2020; Rey et al., 2020). This phenomenon occurs thanks to the forces that the Nichoid provides to cells, thus inducing genetic reprogramming by controlling the cytoskeletal tension (Jacchetti et al., 2021). These results suggest that the Nichoid structure is able to emulate the native stem niche microenvironment in terms of self-renewal and stemness conservation, dictating stem cell fate.

In all the aforementioned studies, proliferation of stem cells inside the polymerized niches was also assessed and compared with that in bidimensional controls, demonstrating the ability of the 3D Nichoid to allow and promote cell expansion.

The aim of this work is to go beyond the examination of mesenchymal stemness, thus interrogating the whole transcriptome of these cells cultured inside the Nichoid, in order to deeper understand gene expression and, thus, better elucidate all MSCs’ physiological functions and therapeutic modes of action (Pittenger et al., 2019). We, thus, performed a complete transcriptome analysis, investigating gene modulation and all the biological functions that are modified by culturing MSCs on 3D Nichoids and comparing them with those of MSCs grown on the recently introduced 2D Nichoid, a substrate consisting of a single layer of polymerized grids that we chose as the bidimensional control since it not only allowed a spreading expansion of cells but also enabled us to evaluate how the pure 3D rearrangement of cells can influence their entire genomic profile.

## 2 Materials and methods

### 2.1 Nichoid fabrication

In this work, we compared Nichoids with two geometries: a single-level pattern (2D Nichoids) and a three-level pattern (3D Nichoids). 2D and 3D micro-patterned substrates were fabricated through 2PP (Raimondi et al., 2012) of a negative hybrid organic–inorganic photoresist named SZ2080 (Ovsianikov et al., 2008) and composed of a 2:8 molar ratio of zirconium propoxide (Sigma-Aldrich, United States) and methacryloxypropil trimethoxysilane (Sigma-Aldrich, United States) (Raimondi et al., 2012; Danilevičius et al., 2013; Ricci et al., 2017). Then, 1% concentration of Irg photoinitiator (Irgacure 369, 2-benzyl-2-dimethylamino-1-(4-morpholinophenyl)-butanone-1) was added to accelerate photopolymerization (Suzuki et al., 2012). A 20 µL drop of resist was deposited on a 12 mm-diameter round glass coverslip suitable for optical microscopy (Bio Optica, Italy) and allowed to harden to a semi-solid state; micropatterns were then fabricated directly on the coverslip. The pulsed laser source employed for fabrication is an ytterbium (Yb):KWY system based on a cavity-dumped oscillator in mode-locking with 1 MHz repetition rate, 1,030 nm wavelength (near-infrared, NIR), and 300 fs pulse duration. The laser beam was focused inside the sample through a 100× magnification oil-immersion microscope objective with 1.4 numerical aperture (NA) (plan apochromat, Carl Zeiss, Germany) and displacement along the three axes controlled by software (Automation 3200 CNC Operator Interface, Aerotech, United States). A spatial light modulator (SLM) was introduced in the setup to speed up the fabrication by splitting the beam into multiple parallel foci (Zandrini et al., 2019). The employed laser power was 100 mW, about 17 mW for each focus of the six-foci mask, and the writing speed was 3 mm/s. There was no slicing in the *z*-direction since it was a single line of 3 µm resolution, while the hatching parameter was 300 nm among two lines of 400 nm, leading to a final resolution of about 1 µm in the xy plane. At the end of the laser writing, the unpolymerized photoresist was removed by leaning the sample in a 50% (v/v) methyl isobutyl ketone and 50% (v/v) isopropyl alcohol solution (Sigma-Aldrich, United States) for 20 min (LaFratta et al., 2007; Malinauskas et al., 2013; Ricci et al., 2017).

The 3D sample had the traditional Nichoid architecture (Ricci et al., 2017): 218 squared blocks made up of 5 × 5 (450 × 450 μm^2^) structures with a spacing of 15 μm (Supplementary Figures S1A, B). Each structure is composed of individual niches, the elementary unit of the structure, with size corresponding to 90 × 90 × 30 μm^3^. Inside, there were three “floors” of interconnected rods, with a graded spacing (10, 20, and 30 μm) in the transverse direction and a uniform spacing of 15 μm in the vertical direction. The 2D Nichoid had the same grid geometry, but since it was made up of only one “floor,” it had an overall thickness of 2 μm, resulting in a partial bi-dimensional scaffold (Supplementary Figures S1C, D) used as a control in all biological experiments.

### 2.2 Substrate preparation and cell seeding

To confine cells inside the surface covered by the polymerized structures and to avoid unwanted cell adhesion outside the Nichoid substrate, different strategies were implemented. First, Costar^®^ 6-well plates with an ultra-low attachment surface (Corning Incorporated, United States) were used as supports for cell seeding both on 3D and 2D Nichoids in order to avoid cell adhesion and proliferation on the surface of the wells. Second, the diameter of the glass sample covered with the Nichoid is 8 mm. To avoid cell adhesion on the glass portion of the coverslip surrounding the patterned portion of the samples, the bottom of the culture wells was drilled to create a small hole, 7 mm in diameter, with respect to the diameter of the Nichoid-covered coverslips. Then, the glass samples covered with the Nichoid were glued to the bottom of the culture wells from below using the biocompatible LOCTITE AA 3321 glue (Henkel, Germany) (Supplementary Figure S2). The glass ring surrounding the Nichoid-covered area of the coverslip corresponded to the glued surface in this assembly. Finally, cells were suspended for seeding in 100 µL of culture medium only, that is, the exact volume needed to guarantee complete covering of the structures only and uniform distribution of cells. This procedure was mainly employed in the case of sample seeded for subsequent RNA extraction in order to limit the cell sample to cells expanded inside the Nichoid structures. For qualitative experiments, such as immunofluorescence image acquisitions, we only used the drop seeding technique, which nevertheless allowed us to ensure controlled disposition of cells on the polymerized structures.

Before seeding, all fabricated samples were sterilized by washing them twice with sterile deionized water, immersing in 70% ethanol for at least 30 min, washing again with sterile water, and letting them dry under a UV lamp (Raimondi et al., 2014).

Primary rat mesenchymal stem cells (rBMSCs), obtained from the bone marrow of Lewis rat and extracted at the Mario Negri Institute for Pharmacological Research (Milano, Italy) following the protocol presented by Zoja et al. (2012), were maintained in culture with an α-minimum essential medium (α-MEM, Gibco, Thermo Fisher Scientific, United States) supplemented by 20% fetal bovine serum (FBS) (EuroClone, Italy), 1% penicillin–streptomycin (EuroClone, Italy), 1% of L-glutamine 200 mM (EuroClone, Italy), and 100 mM of 1% sodium pyruvate (EuroClone, Italy). To avoid differentiation and stemness loss, cells were kept in culture for two passages only and then were detached by trypsin–EDTA solution 1X (EuroClone, Italy), resuspended in phosphate buffered saline (PBS, EuroClone, Italy), and counted using a cell counter (CytoSMART, Netherlands). A total of 3 × 10^4^ cells were seeded on each substrate, and drop-seeded samples were incubated for 1 h to permit proper cell adhesion to the structures; the required volume of medium was then added inside each well.

### 2.3 Viability assay and morphology evaluation

To evaluate viability at the fourth day after seeding, live samples were incubated for 10 min with .5 µm Calcein-AM (Invitrogen, Thermo Fisher Scientific, United States) and .2 µM ethidium homodimer-1 (Invitrogen, Thermo Fisher Scientific, United States) diluted in the fresh culture medium. Calcein-AM distribution was also exploited for a morphological evaluation, and nuclei were stained with 1 µM DRAQ5™ (Thermo Fisher Scientific, United States).

Live fluorescence images were acquired by using the Nikon Ar1+ confocal microscope (Nikon, Japan), equipped with an incubator chamber and four wavelength diode lasers (λ_excitation_ = 405/488/561/640 nm). Stained cells were imaged with a 60× immersion oil objective, with 1.4 NA and .13 working distance (WD). The pinhole was set to 1 Airy unit, and 1,024 × 1,024 pixel images were acquired as z-stack images. Cells grown on glass coverslips and 2D and 3D Nichoids were imaged with a 1 μm step, resulting in an acquisition depth of approximately 10 μm for the first two and a depth of approximately 40 μm for the latter.

After z-stack maximum projections, cellular and nuclear areas were measured through Fiji software v2.3.0/1.53f (Schindelin et al., 2012) by manually drawing cell and nucleus profiles.

### 2.4 RNA data collection

Total RNA was isolated at day 4 using the TRIzol reagent (Total RNA Isolation reagent, Invitrogen, Thermo Fisher Scientific, United States) following the standard protocol (Rio et al., 2010). The extracted RNA was quantified with the Qubit 2.0 Fluorometer (Invitrogen, Thermo Fisher Scientific, United States), and its quality and integrity were evaluated through the Agilent 2100 Bioanalyzer (Agilent Technology, United States). Complementary DNA (cDNA) libraries were prepared using the Universal Plus mRNA-Seq kit (Tecan Genomics, United States) and sequenced in a single-end 75 base pair mode on the NextSeq 500 platform (Illumina, IGA Technologies Services, Italy).

### 2.5 Raw data processing

The per-base quality of the sequenced reads contained in the fastq files was checked with FastQC v0.11.9 (http://www.bioinformatics.babraham.ac.uk/projects/fastqc/). All samples were mapped to the mRatBN7.2 *Rattus norvegicus* reference genome (https://ftp.ensembl.org/pub/release-108/fasta/rattus_norvegicus/dna/), and the BAM files were sorted with STAR v2.7.0a (Dobin et al., 2013). Genes were annotated using standard Ensembl gene annotations, a high-quality annotation system used for vertebrate species that automatically indexes the genomic coordinates of each gene by plotting it onto genome assemblies (Aken et al., 2016). Gene expression levels were assessed by counting aligned reads with HTSeq v2.0.2 (Anders et al., 2015).

### 2.6 Differential expression and functional enrichment analyses

The DESeq2 package of R/Bioconductor (Love et al., 2014) was used to analyze the counts matrix produced by HTseq and to identify differentially expressed genes (DEGs) between rBMSCs grown inside the 3D and 2D Nichoids. As implemented in the DESeq2 package, size factors are estimated to normalize the read counts. Values of |log2(FoldChange)| > 1 and false discovery rate (FDR)-adjusted *p* value ≤ .05 were employed as primary cutoffs to consider expression differences statistically significant.

A functional enrichment analysis to investigate the biological role of DEGs was conducted with the web-based tool g:Profiler (Raudvere et al., 2019) using the Gene Ontology (GO) database. The list of DEGs was ranked by decreasing log2(FC), and an ordered enrichment test was executed. g:Profiler performs Fisher’s exact test to compute the *p* value of the enrichment of a pathway, and multiple-test corrections are applied (Reimand et al., 2019). The cutoff value for significantly enriched pathways was fixed to Benjamini–Hochberg (BH) FDR-adjusted *p* value ≤ .05. The maximum term size was settled at 500.

Subsequently, the GSEA desktop application v4.1.0 (Mootha et al., 2003; Subramanian et al., 2005) was employed to perform a gene set permutation test with default parameters. It takes as input a pre-ranked list made up of all the available genes coming from the expression profile, without applying any cutoff, and uses a permutation-based test aiming to determine whether the genes included in a gene set fall at the top or at the bottom of the ranked list, rather than being randomly distributed within the list (Subramanian et al., 2005). The analysis was carried out with the H (hallmark) and the C2 (curated) gene sets of the Molecular Signatures Database (MSigDB) v7.2 (https://www.gsea-msigdb.org/gsea/msigdb/index.jsp), retrieved and adapted to the *Rattus norvegicus* species through the msigdbr package of R. The number of permutations was settled at 1,000, and an FDR q-value < .25 was chosen as the cutoff for statistical significance.

For a graphical visualization of interactions, a protein–protein interaction network was built with Cytoscape software v3.9.1 (Shannon et al., 2003), and the STRING functional enrichment network plugin was used to generate clusters. As query terms for the network, genes belonging to the most enriched GSEA gene sets were selected.

### 2.7 Quantitative real-time PCR

For validation of selected genes through real-time PCR (RT-PCR), RNA was extracted from 3D and 2D Nichoids as previously described, treated with the DNA-free^™^ DNA removal kit (Invitrogen, Thermo Fisher Scientific, United States), and reverse transcribed into cDNA using the SuperScript^™^ IV VILO^™^ kit (Invitrogen, Thermo Fisher Scientific, United States). Real-time PCR amplification was performed with the StepOnePlus^™^ Real-Time PCR System (Applied Biosystems, Thermo Fisher Scientific, United States) and the GoTaq^®^ qPCR Master Mix (Promega, United States). The following primers (Thermo Fischer Scientific, United States) were designed with NCBI Primer-BLAST and Primer3Plus web tools and cross-checked with the Ensembl gene database: Vcam1 (forward: CTG​TTT​GCA​GTC​TCT​CAA​GC; reverse: AGT​CTC​CAA​TCT​GAG​CGA​GC), Ncam1 (forward: GTA​TGA​TGC​CAA​AGA​AGC​CAA​CA; reverse: TGT​CTT​GAA​CTC​AGT​GGC​TG), Selplg (forward: GGG​GCT​GGA​ACT​TCT​GAG​AC; reverse: CCGTGGGTGCTAGCCG), and Efemp1 (forward: GCT​CCC​CGC​AGG​TAT​CTT​TT; reverse: ATC​GGT​GCA​TTG​CGT​GTA​TG). Gapdh (forward: GGC​AAG​TTC​AAC​GGC​ACA​G; reverse: CGC​CAG​TAG​ACT​CCA​CGA​C) was chosen as the housekeeping gene to normalize samples and calculate the gene expression level following the ∆∆ C_t_ method (Livak and Schmittgen, 2001).

### 2.8 Immunofluorescence assay

For immunofluorescence (IF) staining, samples were washed in PBS (Sigma-Aldrich, United States) and fixed in 10% formalin (Bio Optica, Italy) for 15 min. After three washes in glycine (Sigma-Aldrich, United States) to reduce autofluorescence, cells were permeabilized with PBS–.25% Triton^®^ X-100 (Sigma-Aldrich, United States) for 10 min and blocked in PBS–.1% TWEEN^®^ 20 (Sigma-Aldrich, United States) + 2% FBS (EuroClone, Italy) for 4 h. Primary antibodies to stain YAP (rabbit monoclonal anti-YAP antibody, 1:100, #14074, Cell Signaling Technology, United States) and N-cadherin (mouse anti-cadherin N antibody, 1:200, #94622, Immunological Science, Italy) were introduced and incubated at 4°C overnight. Samples were then washed three times in PBS–.1% TWEEN^®^ 20 and incubated with Alexa Fluor^®^ 647 and Alexa Fluor^®^ 488 (1:750, Abcam, UK) secondary antibodies for 45 min at room temperature. FITC-conjugated phalloidin (Sigma-Aldrich, United States) was added, where needed, to stain the actin cytoskeleton. After three additional washes in PBS–.1% TWEEN^®^ 20, the nuclei were stained with Hoechst 33342 (1:500, Thermo Fisher Scientific, United States) and incubated for 10 min at room temperature. Finally, samples were mounted with Mowiol DABCO^®^ (Sigma-Aldrich, United States) and inspected with a confocal microscope (FLUOVIEW FV10i, Olympus, Italy) equipped with four diode lasers (405, 473, 559, and 635 nm excitation wavelengths). A ×60 water immersion objective with 1.2 NA was used, and the pinhole was set to 1 Airy unit. Z-stack images of around 35 µm depth for 3D Nichoids and 10 µm depth for 2D Nichoids were acquired with a 1 µm step.

Fiji software v2.3.0/1.53f (Schindelin et al., 2012) was used for channel merge and fluorescence intensity quantification.

### 2.9 Statistical analysis

Data were reported as mean values ± standard deviations. The statistical analyses were performed with Student’s *t*-test, and a *p* value ≤ .05 was chosen as the cutoff for significance. **** indicates *p* values ≤ .0001, ** indicates *p* values ≤ .01, and * indicates *p* values ≤ .05.

## 3 Results

As a starting point, we carried out a comparison in terms of viability and morphology among cells cultured on traditional glass coverslips and both the 2D and 3D Nichoids.

**FIGURE 1:**
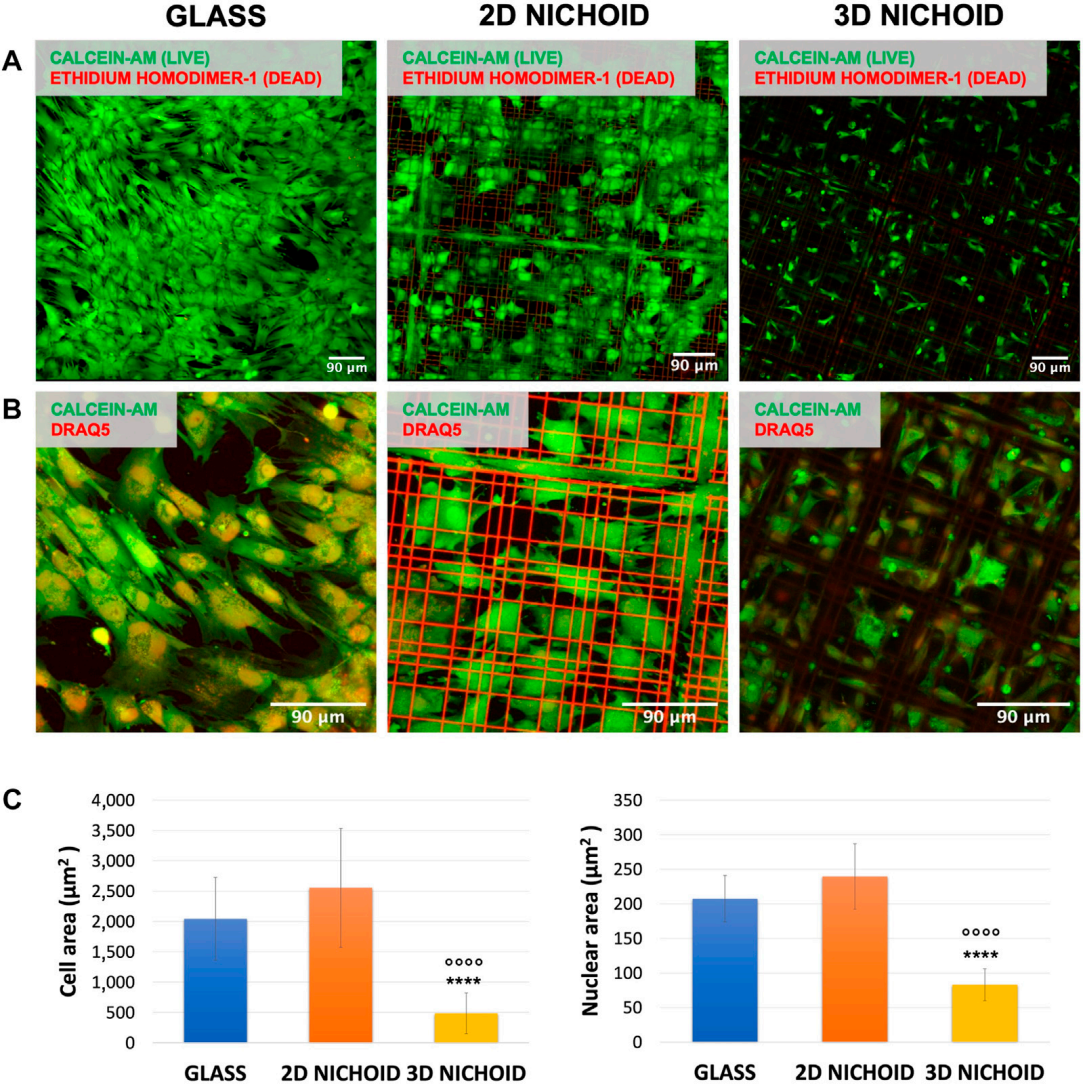
**(A)** Fluorescence images of live (green) and dead (red) rBMSCs cultured on glass coverslips and 2D and 3D Nichoids at day 4. Scale bar = 90 μm. **(B)** Fluorescence images of the cytoplasm (green) and nuclei (red) of rBMSCs cultured on glass coverslips and 2D and 3D Nichoids at day 4. Scale bar = 90 μm. **(C)** Quantification of the cellular and nuclear area in the three conditions. **** indicates *p* value ≤ .0001 for the 3D vs. 2D Nichoid comparison, and ^oooo^ indicates *p* value ≤ .0001 for the 3D vs. glass comparison.

rBMSC viability was assessed after 4 days of culture, the same time point of RNA extraction used for sequencing analysis. At least three areas per sample were acquired, and a strong predominance (close to 100%) of vital cells (in green) with respect to apoptotic cells (in red) was observed on all the used substrates (Figure 1A).

On the same samples, morphology analyses were performed, and the fluorescent dye DRAQ5 was added to also visualize the cell nucleus. Our observations revealed a variation between the three substrates in terms of cell shape. As visible in Figure 1B, cells cultured on 2D Nichoids were wide and spread, with morphology and dimension more comparable to those grown on glass coverslips, whereas in 3D Nichoids, cells tended to be more retained and confined. The quantification of cellular areas confirmed that cells inside the 3D environment are significantly smaller than those in both of the two flat substrates, whereas among the 2D Nichoid and glass coverslips, no discrepancy emerged. In addition, the quantification of nuclear areas highlighted a significant reduction in the dimensions of 3D-grown cells’ nuclei with respect to the other conditions (Figure 1C).

Based on these observations, we could state that rBMSCs cultured on 2D Nichoids had a bidimensional and spreading expansion compared to cells growing on glass coverslips. For this reason, we held it reasonable to employ a 2D Nichoid as a flat control in all subsequent experiments.

### 3.1 YAP nuclear/cytoplasmic localization

**FIGURE 2:**
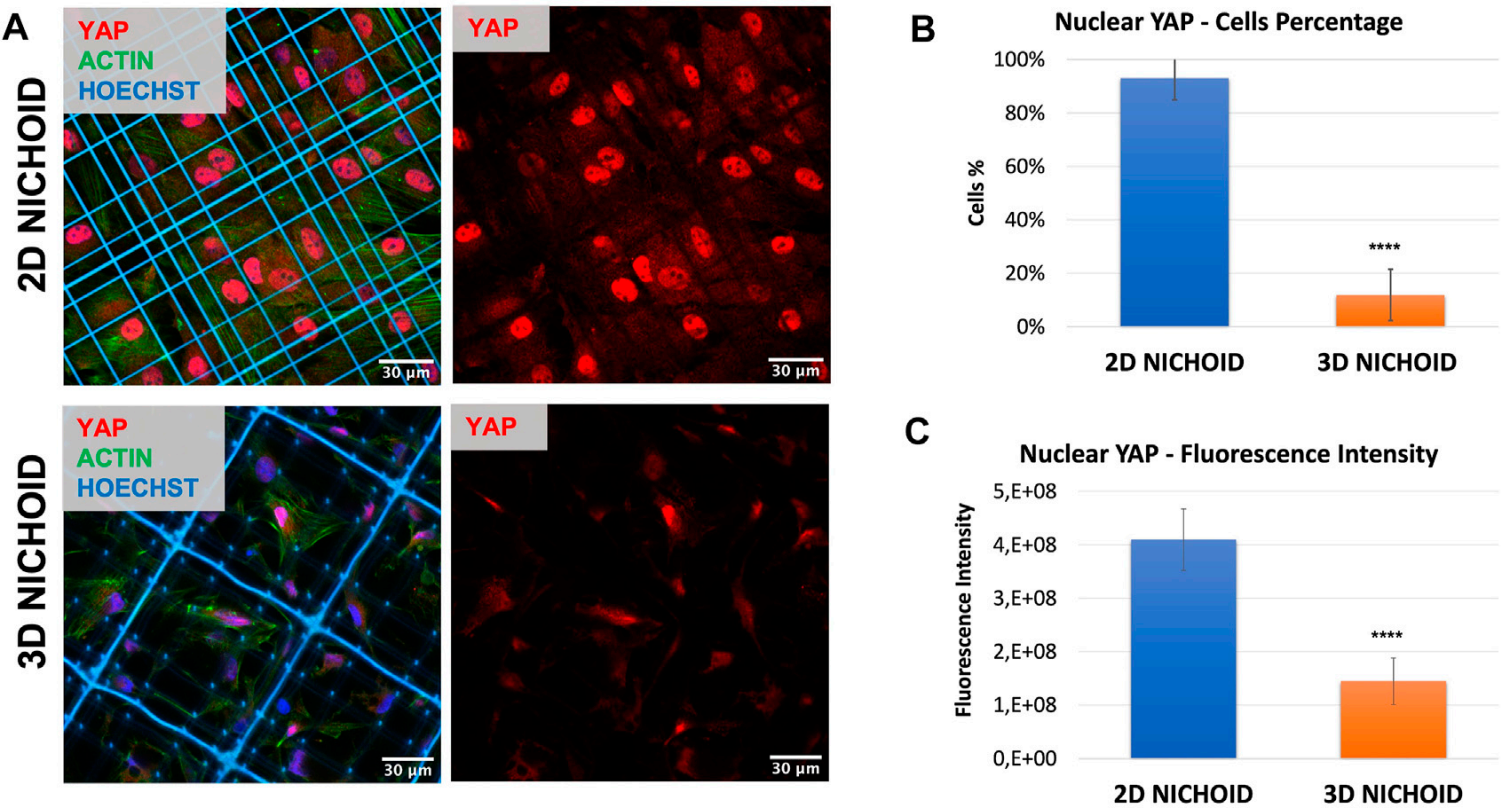
**(A)** Immunofluorescence images of rBMSCs with stained nuclei (blue), actin (green), and YAP (red) in 3D and 2D Nichoids. Scale bar = 30 µm. **(B)** Quantification of the rBMSC percentage of cells with YAP mainly localized in the nucleus; **** indicates *p* value ≤ .0001 for the 3D vs. 2D Nichoid comparison (*n* = 4). **(C)** Quantification of rBMSCs’ fluorescence intensity of nuclear YAP; **** indicates *p* value ≤ .0001 for the 3D vs. 2D Nichoid comparison (*n* = 20).

To corroborate the possibility of using a 2D Nichoid instead of a glass coverslip, we stained the mechano-transducer factor yes-associated protein (YAP) by immunofluorescence to compare its localization in bidimensional and tridimensional culture conditions. Immunofluorescence images (Figure 2A) clearly displayed that, just as it happened on glass coverslips (Remuzzi et al., 2020), in rBMSCs cultured on 2D Nichoids, YAP was almost exclusively localized in the nucleus, while it consistently translocated in the cytoplasm when cells were grown in a 3D environment. Indeed, on average, 93% of cells in 2D Nichoids retained YAP mostly inside the nucleus, whereas in 3D Nichoids, this percentage was around 12% (Figure 2B), and the YAP fluorescence level was significantly higher in the nuclei of cells cultured on bidimensional controls (Figure 2C).

### 3.2 Differentially expressed genes identification and functional enrichment

**FIGURE 3:**
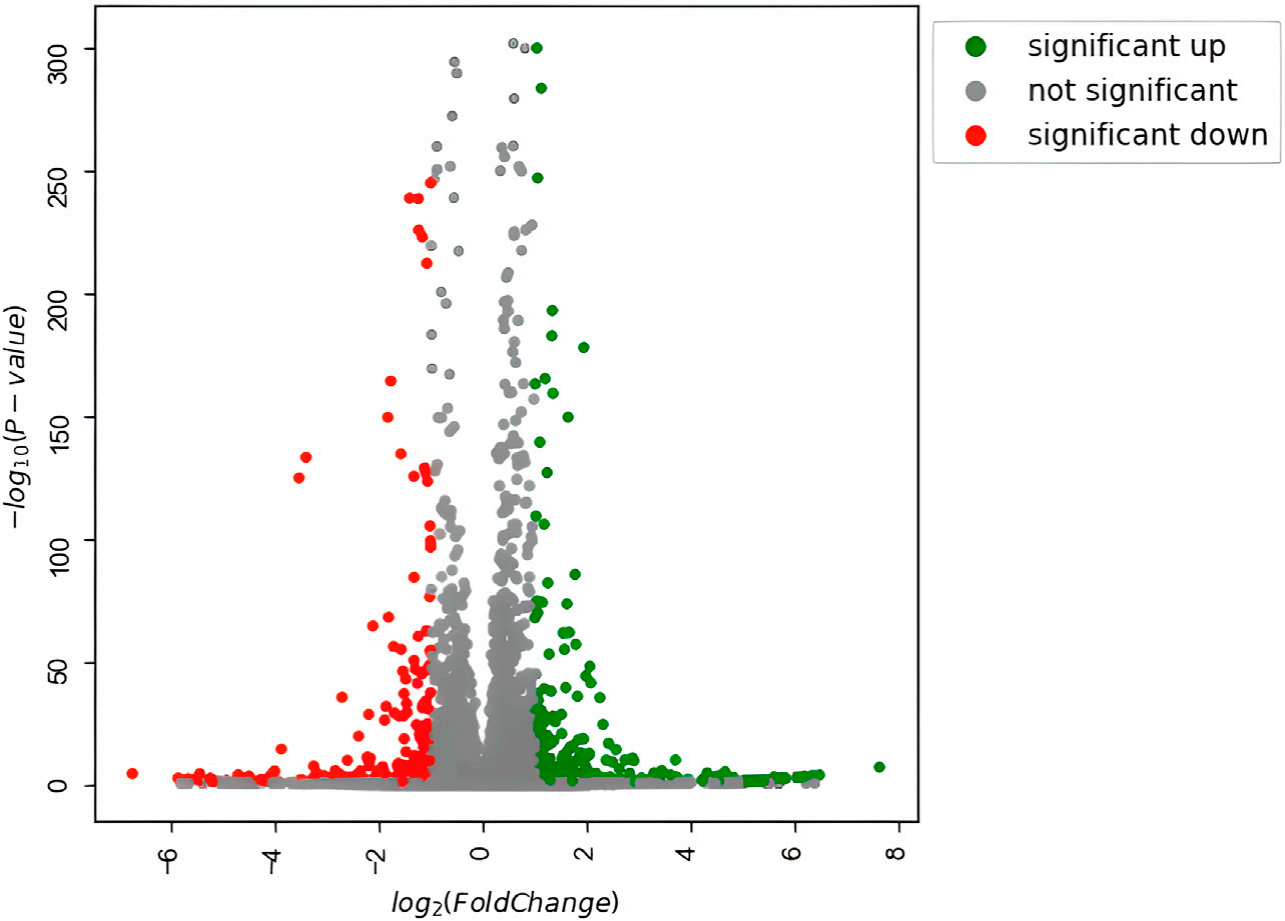
Volcano plot of differentially expressed genes. Significantly upregulated genes [adjusted *p* value ≤ .05 and log2(FC) > 1] are in green; significantly downregulated genes [adjusted *p* value ≤ .05 and log2(FC) < 1] are in red.

To investigate how the previously observed morphological differences generated by Nichoid three-dimensionality translate into variations in terms of rBMSC gene expression, the profiling of the complete transcriptome of Nichoid cultures was performed. All genes resulting from the total RNA-seq are reported in Supplementary Table S1. Results from the bioinformatic pipeline showed that at 4 days from cell seeding, gene deregulation was affected in a significant way, since 654 genes appeared to be differentially expressed (392 upregulated and 262 downregulated) between 3D and 2D Nichoids, which are listed in Supplementary Table S2. All DEGs, defined as those genes that meet both |log2(FoldChange)| > 1 and false discovery rate (FDR)-adjusted *p* value ≤ .05 cutoffs, are depicted in green (upregulated) and red (downregulated) in the volcano plot in Figure 3.

**FIGURE 4:**
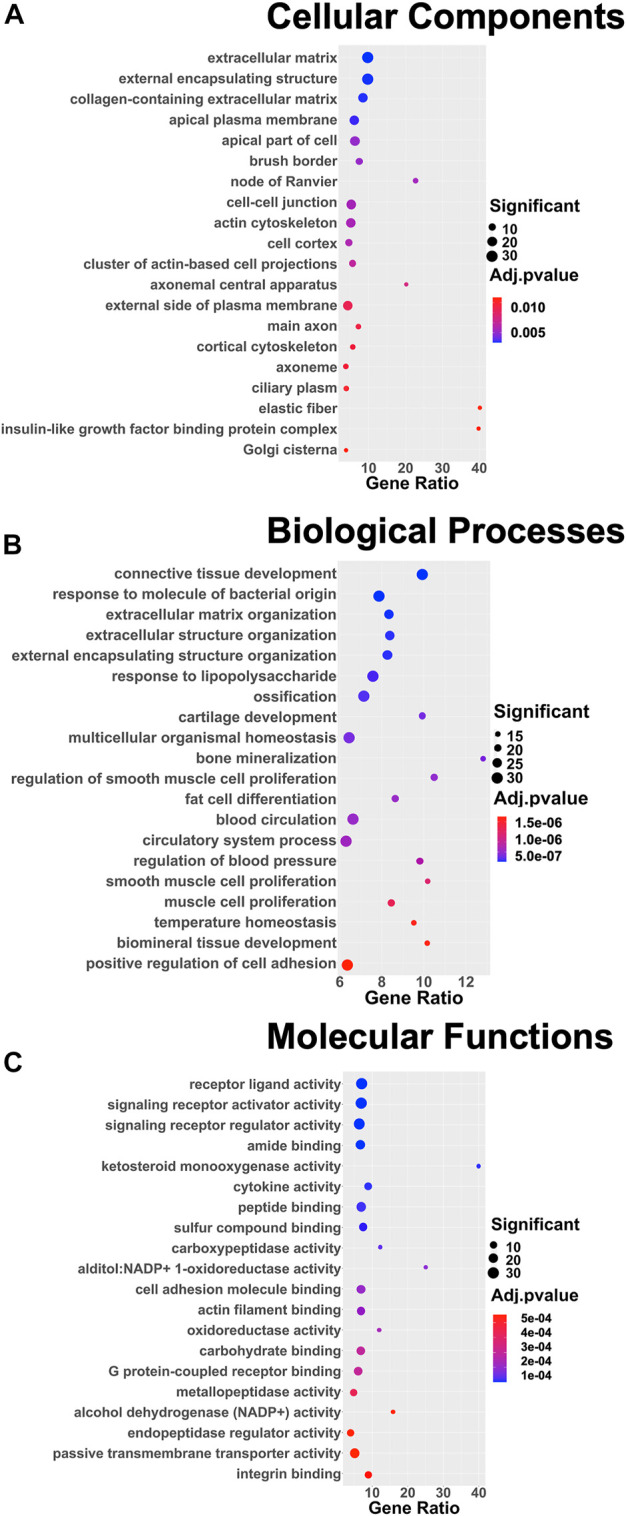
Dot plots of the 20 most enriched terms of the three different GO categories: **(A)** cellular components, **(B)** biological processes, **(C)** and molecular functions. The color of the dot represents the adjusted *p* value according to the color scale on the right side of the plots; the dimension of the dot represents the number of significant DEGs involved in the pathway; and the position of the dot on the *x*-axis represents the fraction of DEGs on the total number of genes belonging to the pathway.

DEGs were successively used as input datasets for a biological enrichment analysis with the GO database to examine in which pathways they are mainly enriched. Interestingly, the highest dysregulated cellular components category has an extracellular matrix (GO:0031012), in particular, collagen-containing extracellular matrix (GO:0062023), apical plasma membrane (GO:0016324), apical part of the cell (GO:0045177), cell–cell junction (GO:0005911), actin cytoskeleton (GO:0015629), cluster of actin-based cell projections (GO:0098862), external side of the plasma membrane (GO:0009897), and cortical cytoskeleton (GO:0030863). The extracellular space appears to be dysregulated also in the biological functions category [extracellular matrix organization (GO:0030198) and extracellular structure organization (GO:0043062)], together with the regulation of cell adhesion (GO:0045785) and a series of pathways related to the muscle (GO:0033002; GO:0048659), bone (GO:0001503), cartilage (GO:0051216), and adipose tissue (GO:0045444) lineages. Moreover, in the molecular functions category, the top deregulated pathways are related to receptor activity (GO:0030546), cytokine activity (GO:0005125), cell adhesion molecule binding (GO:0050839), actin filament binding (GO:0051015), and integrin binding (GO:0005178) (Figure 4).

**FIGURE 5:**
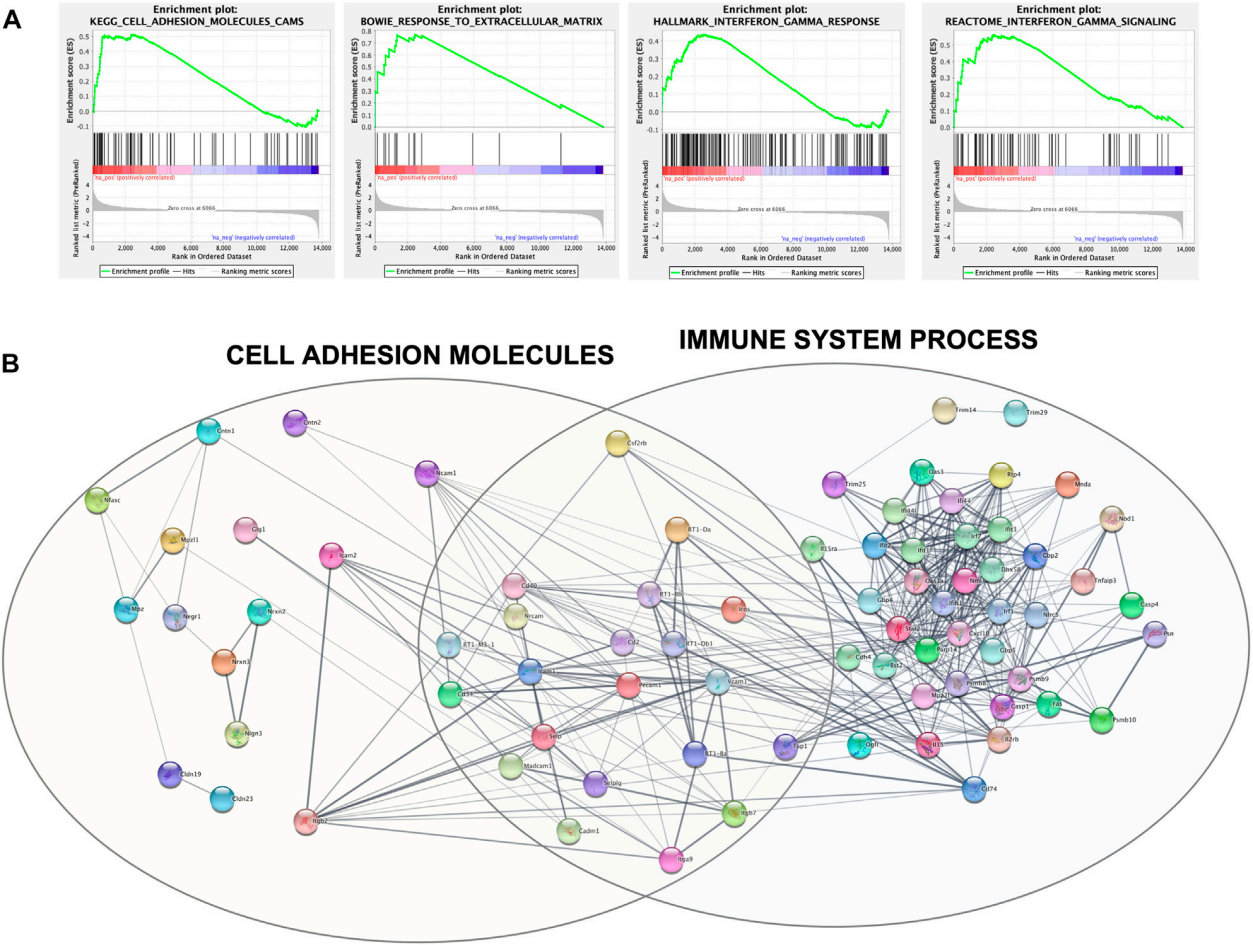
**(A)** GSEA output of the four mainly enriched gene sets. Each vertical black line corresponds to a gene, and its shift to the left indicates its positive enrichment. **(B)** Protein–protein interaction network built with Cytoscape and clustered with the STRING functional enrichment network plugin.

For a deeper enrichment investigation, we exploited the GSEA approach. This analysis also showed, among all the aforementioned pathways, significant and marked modifications in the expression of genes related to cell adhesion molecules and the extracellular matrix. Indeed, the KEGG_CELL_ADHESION_MOLECULES_CAMS and BOWIE_RESPONSE_TO_EXTRACELLULAR_MATRIX gene sets had positive enrichment scores, ES = .51 and ES = .77, respectively, meaning that they were significantly (FDR q-value = .24 and FDR q-value = .17) overrepresented at the top of the ranked list of genes in the expression dataset. This is also graphically visible by the majority of black lines belonging to the gene set moved to the left. Interestingly, other positively and significantly enriched gene sets were REACTOME_INTERFERON_GAMMA_SIGNALING (ES = .56; FDR q-value = .14) and HALLMARK_INTERFERON_GAMMA_RESPONSE (ES = .44; FDR q-value = .002) (Figure 5A).

The set of genes belonging to these four significantly enriched gene sets was used as query terms for network construction to investigate potential connections. The network was divided into several clusters; the main two are shown in Figure 5B and include core genes with the highest rank metric scores of CAMs and IFN- γ gene sets. Genes belonging to the response to the ECM gene set are contained in the IFN- γ cluster. This graphical visualization highlighted how these pathways are closely interconnected with each other.

### 3.3 Cell adhesion molecules

**FIGURE 6:**
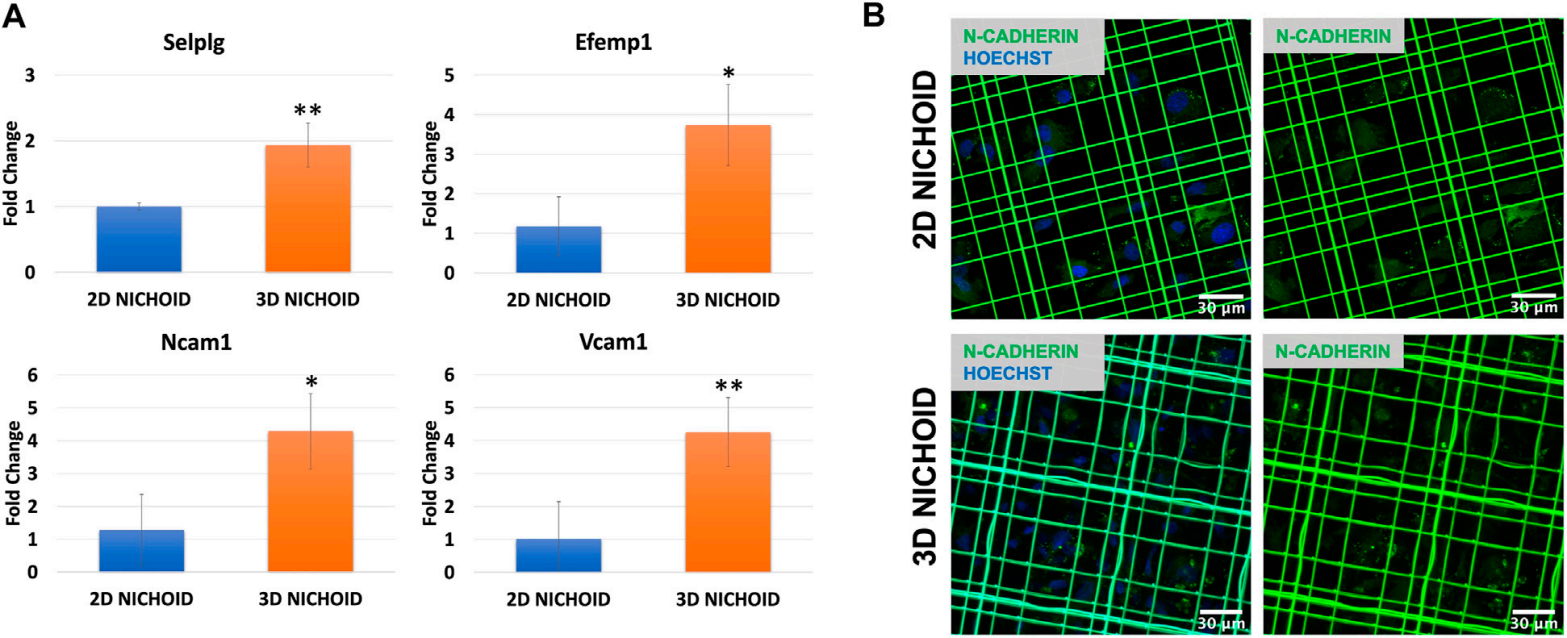
**(A)** Gene expression quantification through real-time PCR of Selplg (*n* = 3), Efemp1 (*n* = 4), Ncam1 (*n* = 3), and Vcam1 (*n* = 4) in rBMSCs grown in 3D and 2D Nichoids for 4 days. Gapdh was used as the housekeeping gene. Data are represented as mean ± SD; * indicates *p* value ≤ .05, and ** indicates *p* value ≤ .01 vs. 2D Nichoid. **(B)** Immunofluorescence images of nuclei (blue) and N-cadherin (green) in 3D and 2D Nichoids are shown. Scale bar = 30 µm.

We then focused our attention on adhesion molecules. Therefore, the expression of four upregulated genes belonging to enriched pathways was analyzed through RT-PCR, which confirmed the overexpression of Selplg, Vcam1, Ncam1, and Efemp1 detected from the sequencing in the 3D Nichoid with respect to the 2D Nichoid (Figure 6A).

To also evaluate homotypic cell–cell adhesions, we stained N-cadherin, a calcium-dependent cell adhesion protein that is predominantly expressed by MSCs (Hatta and Takeichi, 1986; Wuchter et al., 2007). Immunofluorescence images display a slight increase, although not significant, in the synthesis of this protein in rBMSCs grown in 3D Nichoids compared to bidimensional expansion. More interestingly, images showed a different localization of the proteins inside cells: in rBMSCs grown in 2D Nichoids, N-cadherin appears to be homogeneously distributed in the cytoplasm, whereas in 3D Nichoids, it is more localized in spots and distant from the nucleus in areas of cell–cell contacts (Figure 6B).

## 4 Discussion

Mesenchymal stem cells appear to be among the most promising stem cell sources for cell-based regenerative therapies, thanks mainly to their ease of extraction from several tissues and their differentiation potential toward a wide cohort of end-stage lineages (Han et al., 2019). Nevertheless, beyond their potential for differentiation and tissue regeneration, it has been demonstrated that MSCs have immunomodulatory and anti-inflammatory properties exercised through cytokine secretion and trophic activity (Zhao et al., 2016; Weiss and Dahlke, 2019; Song et al., 2020); they express a large number of genes encoding for a variety of regulatory proteins involved not only in mesoderm specification but also in other biological processes including inflammation and immune activation, cell motility, and communication (Phinney et al., 2006; Phinney, 2007; Phinney, 2009; Cruz-Barrera et al., 2020). Furthermore, it has been demonstrated that among the mostly expressed transcripts, there are also those encoding for structural and functional extracellular matrix (ECM) proteins, which may contribute to MSCs’ clinical regenerative potential, immune modulatory effects, and anti-inflammatory effects (J. Ren et al., 2011). These features make MSCs an ideal source for autologous cell-based treatments since their trophism could be even more significant for their therapeutic potential than their pluripotency (Phinney, 2007; Lukomska et al., 2019).

However, to enable these cells to maintain all their characteristics and properties, it is of fundamental relevance to expand them in an appropriate environment where they can replicate *in vitro* the correct disposition and conformation they possess *in vivo* through the restoration of their physiological shape and the proper interactions with both the ECM and surrounding cells. In order to model a more physiological environment than the one that can be obtained with a simple slide or plate, we employed the Nichoid, a 3D scaffold designed to replicate the architectural microenvironment of the native stem niche. It is fabricated through a two-photon laser polymerization technique, the only one capable of achieving a defined structure at the sub-micrometric level, which is essential to ensure control over MSC responses at the individual cell scale.

Cells cultured on Nichoid samples were first examined to assess morphological and viability differences between three culture conditions. In 2D Nichoids, cells adhered to the underlying glass, and they showed a flattened and spread shape, correlated to an anisotropic tensional state, with an area comparable to that of cells cultured on traditional glass coverslips. On the contrary, in 3D Nichoids, the cytoplasm of cells was distributed in a more confined space, and evidence from several sources in the literature shows that this morphology is linked to reduced cytoskeletal tensions on the nucleus (Jacchetti et al., 2021) and to a greater cell multipotency (Nava et al., 2012; Saei Arezoumand et al., 2017). Despite these morphological differences, the viability assay did not show a significant difference, confirming the biocompatibility of the employed resin and demonstrating that the scaffold material in both 3D and 2D substrates does not impact rBMSC viability. These inspections allowed substituting the standard glass coverslip as the bidimensional control with the 2D Nichoid in all experiments, allowing us to determine the mere architectural effect of the scaffold tridimensionality on rBMSC gene expression and protein localization, separating it from the influence of the material properties relevant to the scaffold. In support of this hypothesis, our immunofluorescence results confirmed the same behavior observed by Remuzzi et al. (2020), where an increased percentage of the cytoplasmic fraction of YAP in rBMSCs cultured in 3D Nichoids was appreciable with respect to rBMSCs grown in monolayers on flat controls. In particular, this occurred despite differences in the number of cells seeded, time points, and, above all, substrate used as the flat control, which, in our case, was the 2D Nichoid in place of glass coverslips.

To investigate the consequent variation in gene expression inside the two different culture substrates, the transcriptome of rBMSCs cultured on Nichoids was sequenced and analyzed, aiming at determining the “niche effect” provided by the engineered cues of the 3D Nichoid architecture on the whole stem cell genetic response. For this purpose, the total RNA extracted from rBMSCs cultured on 2D and 3D Nichoids was profiled for the first time using next-generation sequencing technology, which allows for a more straightforward and affordable analysis of gene regulation compared to microarrays and other sequencing techniques with a higher sensitivity in detecting differentially expressed genes (J. Li et al., 2016; Marguerat and Bähler, 2010).

The measured differential expression confirmed that the morphological differences induced by the two substrates are enough to lead to a significant genetic reprogramming of cellular processes, and the experimental design in use leads to the conclusion that these results are attributable exclusively to the three-dimensionality of the culture substrate.

To understand in which pathways they were mainly involved, the identified DEGs were subsequently subjected to functional enrichment for GO annotation with g:Profiler. The list of the top 20 affected pathways in the three categories seems to suggest that there was a deregulation in the entire mechano-transduction chain, starting from the extracellular matrix, moving through cell adhesions, and, by means of a reorganization of the actin cytoskeleton, reaching the nucleus. The modifications in this chain influenced biological processes and molecular functions of rBMSCs that were different between the 3D and 2D Nichoids, which mainly involved differentiation toward different lineages, receptors, and cytokine activity. The GSEA further underlined the discrepancy between the two culture systems in the expression of genes encoding for cell adhesion molecules and in the response to the ECM. This analysis revealed that two gene sets related to IFN- γ also appeared to be upregulated, and interestingly, several connections were identified among the four gene sets by network analysis, reiterating how CAMs and the ECM play a relevant role also in the response of immune-related functions. These pathways are relevant since it is well established that MSCs primed with IFN- γ enhance their immunosuppressive capacity (Klinker et al., 2017; Kim et al., 2018). However, several pieces of evidence showed that 3D-disposed MSCs are capable of self-activating their paracrine functions, increasing their pro-angiogenic potential, and intensifying the secretion of factors associated with immune cell modulation and tissue regeneration, even without external inflammatory signals (Ylostalo et al., 2014; Murphy et al., 2017; Xie et al., 2021).

Following the results obtained from the enrichment analyses, we focused our attention on this gene set, confirming the upregulation of Selplg, Vcam1, Ncam1, and Efemp1 in the 3D Nichoid with respect to the 2D Nichoid *via* RT-PCR experiments. Selplg encodes for the P-selectin glycoprotein ligand-1, which is part of the adhesion molecules involved in the regulation of leukocyte migration in response to inflammatory stimuli (Hirata et al., 2000; Hidalgo et al., 2007); interestingly, it has been shown how MSCs expressing this gene can rapidly move to the site of inflammation, exerting a superior anti-inflammatory effect (Levy et al., 2013; Liao et al., 2016). In addition to being an MSC marker, Vcam1 (vascular cell adhesion molecule 1) is recognized to play a critical role in MSC-related immunosuppression, and its expression is induced by the simultaneous presence of IFN- γ and inflammatory cytokines (Ren et al., 2010). Moreover, Ncam1 (neural cell adhesion molecule 1) has been found to be expressed in MSCs and involved in cell migration *via* MAPK/ERK signaling activation (Shi et al., 2012). Efemp1, also known as fibulin-3, is a gene that encodes for the EGF-containing fibulin-like extracellular matrix protein 1, which is involved not only in cell adhesion and migration but also in negative regulation of chondrocyte differentiation (GO:0032331). Vukovic et al. (2009) demonstrated that this protein is highly expressed in olfactory ensheathing cells (OECs), which are endowed with a noteworthy self-repair potential and, together with their associated ECM, play an important role in proliferation during regenerative events. These results indicated that one of the effects of the 3D Nichoid is to regulate the adhesion molecules related to the immunomodulatory pathways; therefore, one might reasonably assert that the 3D Nichoid is able to affect the immunomodulatory ability of MSCs.

The upregulation of Vcam1 in 3D Nichoids could also be related to a higher number of cell–cell contacts mediated by N-cadherin that we investigated through immunofluorescence staining. It has been demonstrated that cell–cell adhesion mediated by N-cadherin promotes the expression of Vcam1 by activating the nuclear factor κB (NF-κB) pathway *via* the platelet-derived growth factor receptor beta (PDGFRβ) (Aomatsu et al., 2014; Chosa and Ishisaki, 2018). Our results show that this contact protein was slightly more expressed in 3D Nichoids, but above all, it was more aggregated; this suggests that the 3D structure of the scaffold promoted MSC-homotypic cell–cell adhesion among neighboring cells.

Cell–cell adhesions through N-cadherin also underlay the translocation of the mechano-transduction factor YAP from the nucleus to the cytoplasm. YAP, together with the transcriptional co-activator with a PDZ-binding motif (TAZ), is a key driver of stem cell behavior since it responds to physical stimuli, such as ECM stiffness, cell geometry, or mechanical forces of the cytoskeleton, with specific transcriptional programs (Donnaloja et al., 2020; Y. Li et al., 2021; Piccolo et al., 2014). As a matter of fact, the spatial organization of focal adhesions in cells inside the 3D Nichoid modifies the transmission of forces to the nucleus, determining weaker forces than in spreading cells and, thus, affecting the nuclear import of signaling molecules (Jacchetti et al., 2021). In addition to the effect of focal adhesions, the presence of N-cadherin, which is involved in mechano-transduction, affects the perception of the microenvironment stiffness, reducing the contractile state of the cell and subsequently YAP translocation (Cosgrove et al., 2016; Qin et al., 2020; Zhang et al., 2021).

YAP is a fundamental downstream effector of the Hippo pathway, which plays a role in several mechanisms such as development, stem cell self-renewal and differentiation, regeneration, immune modulation, and cancer (Moya and Halder, 2019; Dey et al., 2020; LeBlanc et al., 2021). Among target genes of activated YAP/TAZ, in our transcriptome analysis, we found both Snai2 (zinc finger protein SNAI2) and Jag1 (protein jagged-1) to be overexpressed in 3D Nichoids. It has been proven that Snai2 is a transcription factor included among the major upregulators for BM–MSC maintenance, fate decision, cell adhesion, and cell structure regulation (Sánchez-Luis et al., 2020; Wei et al., 2020). In turn, Snai2 can bind to Cxcl12 (C-X-C motif chemokine 12) promoters; Cxcl12, which we also found slightly over-expressed, is critical to retain both MSCs and hematopoietic stem cells in the bone marrow niche and also for hematopoietic stem and progenitor cells’ quiescent state maintenance (Roson-Burgo et al., 2016). Interestingly, Giri et al. (2020) also demonstrated that BM–MSC-secreted Cxcl12, cooperating with CCL2 (C-C motif chemokine 2), exercised an anti-inflammatory capacity in toxic colitis by upregulating IL10 expression in CCR2^+^ macrophages. Furthermore, Jag1, which is part of the Notch pathway, has been shown to be a potent pro-angiogenic regulator (Benedito et al., 2009).

To conclude, our results demonstrated how restoring both the correct morphology of the stem cells and the correct adhesions in an *in vitro* culture system is important to preserve the native cell functionality. It is known that adhesion molecules are the essential initial point of a mechano-transduction chain that starts from the membrane and, through the cytoskeletal organization and tension, reaches the nucleus, conditioning several biological aspects of the cell, such as transcription. In this context, the Nichoid proved to be able to perform these tasks and, thus, demonstrates, thanks to its peculiar capability to restore 3D cell adhesions, especially those among neighboring cells, to be not only an ideal substrate for MSC expansion and stemness maintenance but also a means for controlling other specific MSC features such as immunomodulation, thus supporting its potential for clinical application in immune-based diseases.

## Data Availability

The data presented in the study are deposited in the GEO repository, accession number GSE199846: https://www.ncbi.nlm.nih.gov/geo/query/acc.cgi?acc=GSE199846.
