# Supplementary figures and images for: Whole transcriptomic analysis of mesenchymal stem cells cultured in Nichoid micro-scaffolds
Source: Front Bioeng Biotechnol. 2023 Jan 6;10:945474. doi: 10.3389/fbioe.2022.945474 (PMC9852851; doi:10.3389/fbioe.2022.945474)

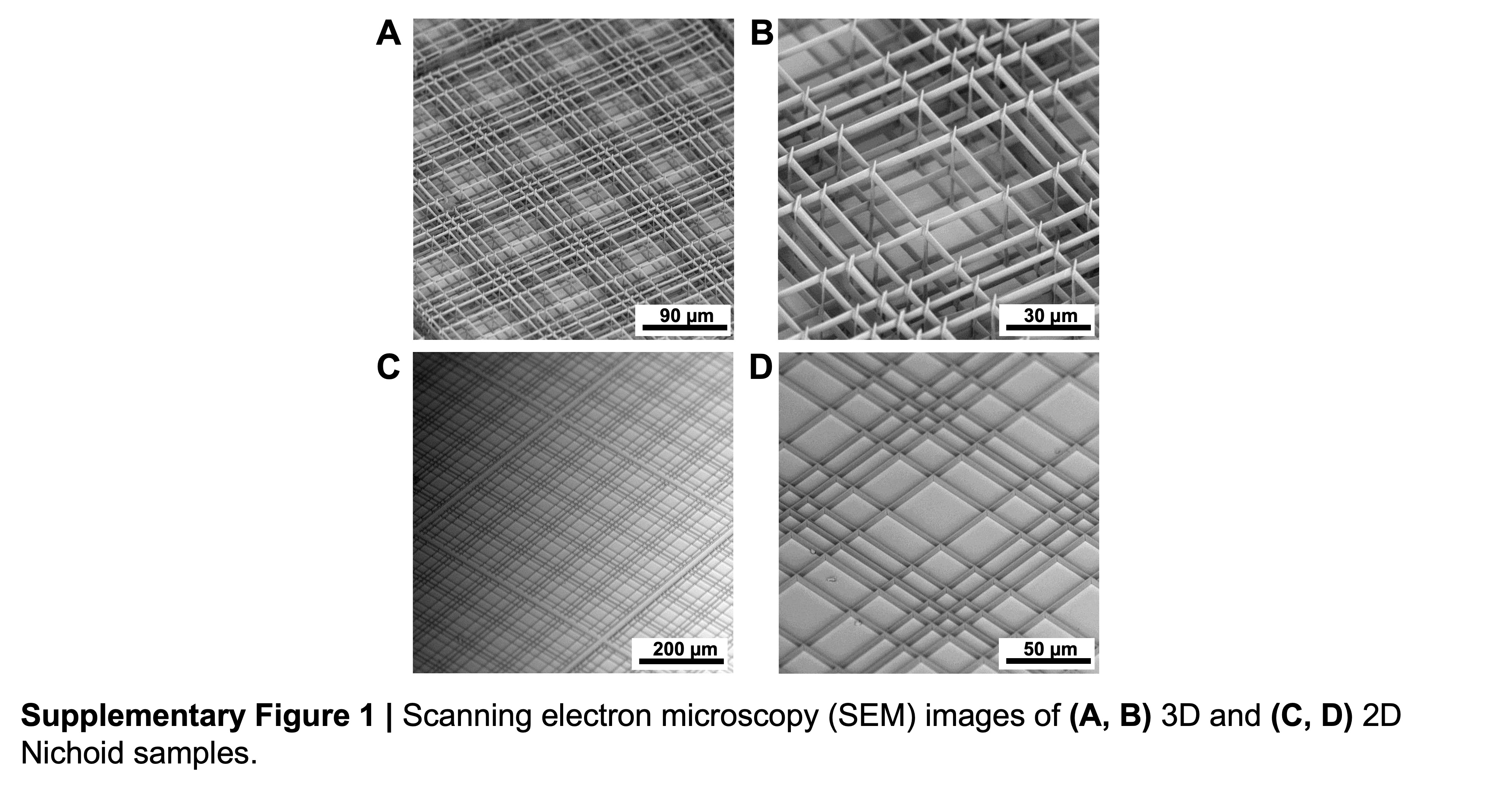

Supplement: Supplementary file 1 [file Image1.JPEG]

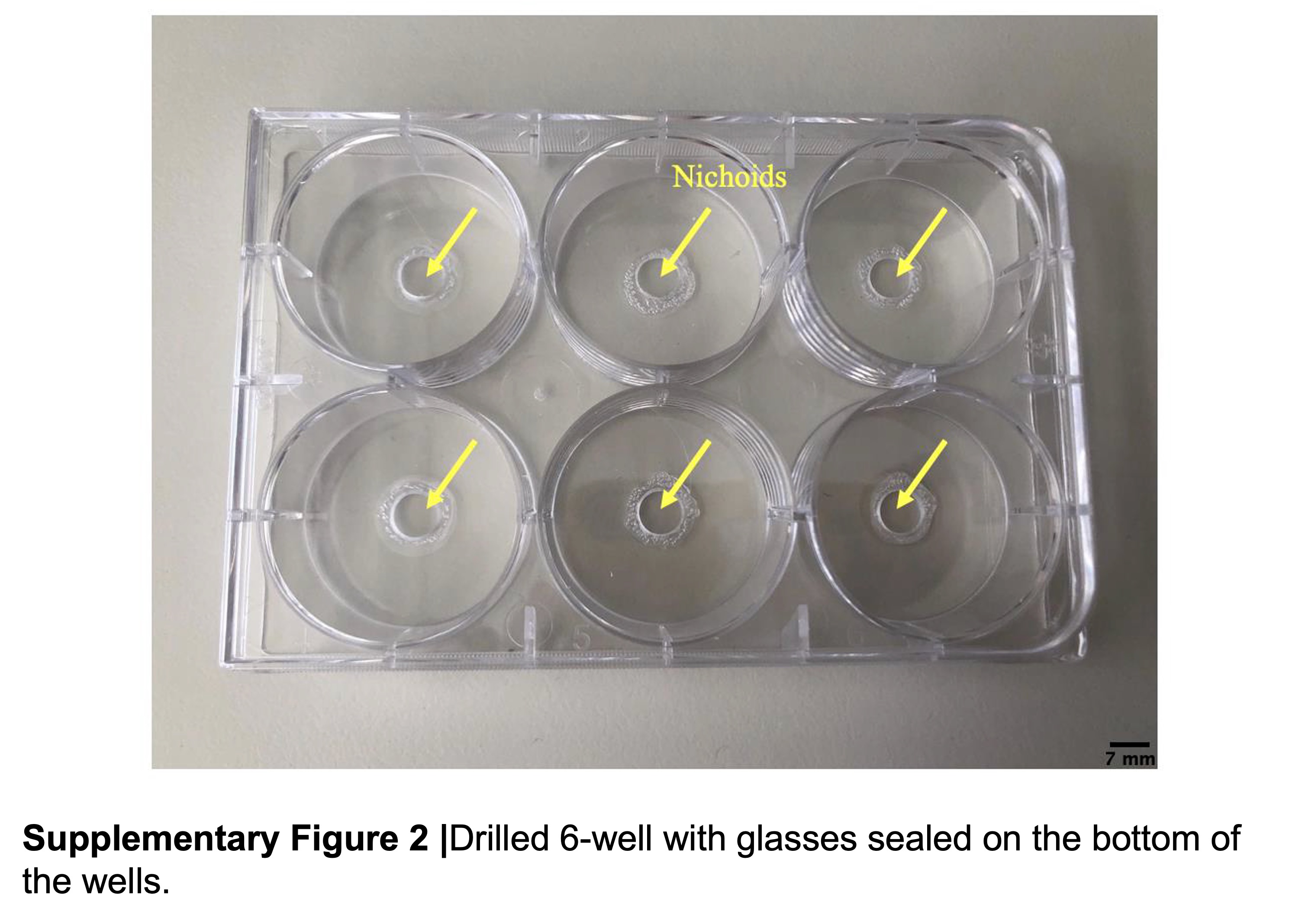

Supplement: Supplementary file 2 [file Image2.JPEG]
